# Neuropeptidergic integration of behavior in *Trichoplax adhaerens*, an animal without synapses

**DOI:** 10.1242/jeb.162396

**Published:** 2017-09-15

**Authors:** Adriano Senatore, Thomas S. Reese, Carolyn L. Smith

**Affiliations:** 1 University of Toronto Mississauga, Mississauga, ON, Canada L5L 1C6; 2 NINDS, NIH, Bethesda, MD 20892, USA

**Keywords:** Endomorphin, Neuropeptide, Cilia, Neurosecretory cells, Nervous system evolution, Placozoa

## Abstract

*Trichoplax adhaerens* is a flat, millimeter-sized marine animal that adheres to surfaces and grazes on algae. *Trichoplax* displays a repertoire of different feeding behaviors despite the apparent absence of a true nervous system with electrical or chemical synapses. It glides along surfaces to find food, propelled by beating cilia on cells at its ventral surface, and pauses during feeding by arresting ciliary beating. We found that when endomorphin-like peptides are applied to an animal, ciliary beating is arrested, mimicking natural feeding pauses. Antibodies against these neuropeptides label cells that express the neurosecretory proteins and voltage-gated calcium channels implicated in regulated secretion. These cells are embedded in the ventral epithelium, where they comprise only 4% of the total, and are concentrated around the edge of the animal. Each bears a cilium likely to be chemosensory and used to detect algae. *Trichoplax* pausing during feeding or spontaneously in the absence of food often induce their neighbors to pause as well, even neighbors not in direct contact. Pausing behavior propagates from animal to animal across distances much greater than the signal that diffuses from just one animal, so we presume that the peptides secreted from one animal elicit secretion from nearby animals. Signal amplification by peptide-induced peptide secretion explains how a small number of sensory secretory cells lacking processes and synapses can evoke a wave of peptide secretion across the entire animal to globally arrest ciliary beating and allow pausing during feeding.

## Abstract (precis)

**Highlighted Article:** *Trichoplax*, a primitive marine animal that lacks neurons and synapses, has neurosecretory cells that secrete a peptide into the ambient seawater to arrest ciliary beating during feeding.

## INTRODUCTION

Placozoans are disk-shaped animals, up to several millimeters in diameter, that adhere to surfaces in littoral zones of tropical and subtropical seas (Schulze, 1883; Grell and Ruthmann, 1991; Schierwater, 2005). *Trichoplax adhaerens* is the only named member of the phylum, and has been the focus of most research on placozoans. When maintained in the laboratory with microalgae as a food source, *Trichoplax* locomotes by gliding on cilia arising from its ventral epithelial cells, and periodically pauses to externally digest algae (Smith et al., 2015).

*Trichoplax* has a simple body plan with no axis of symmetry and just six identified cell types (Smith et al., 2014). The ventral epithelium is thick and columnar, and comprises three of these cell types. Approximately 70% of the cells in the ventral epithelium are typical monociliated epithelial cells. Their cilia beat asynchronously to propel gliding and cease beating to arrest movement during feeding. Evenly interspersed among the ciliated ventral epithelial cells are lipophil cells, which contain large lipophilic granules that are secreted to externally digest algae. The third cell type, a secretory cell containing smaller granules, is dispersed mainly around the periphery of the ventral epithelium. These secretory cells contain the machinery for calcium-regulated secretion, secretory SNARE proteins (syntaxin, synaptobrevin, SNAP-25; Smith et al., 2014) and complexin (Smith et al., 2017), as well as voltage-gated calcium channels (Ca_V_2, Ca_V_3; Senatore et al., 2016; Smith et al., 2017). Their granules label for RFamides (Schuchert, 1993; Smith et al., 2014), a class of neuropeptides that is common in cnidarians and bilaterians and is implicated in the control of ciliary beating (Katsukura et al., 2004; Braubach et al., 2006; Conzelmann et al., 2011). They each also bear a single cilium that could be chemosensory and used to detect algae.

Ciliated sensory secretory cells are common in the ciliated epithelia of ctenophores, cnidarians and bilatarians (Harrison and Westfall, 1991; Mitchell, 2007, 2016; Tamm, 2014). Ciliated cells containing secretory-like vesicles also are present in poriferan larvae (Nakanishi et al., 2015). Sensory cilia often lack the central pair of microtubules typical of motile cilia and may be either motile or immotile (Ludeman et al., 2014; Singla and Reiter, 2006). Choanoflagellates, unicellular or colonial protists that are the closest sisters to the metazoans, have a cilium that is motile and possibly also sensory, mediating responses to chemical cues in the environment (Alegado et al., 2012; Woznica et al., 2016). Furthermore, choanoflagellates express secretory SNARE proteins and contain granules resembling the secretory granules of metazoan secretory cells (Burkhardt et al., 2011).

All the cells in the ventral epithelium of *Trichoplax* are joined by adherens junctions, as are cells in the thinner, dorsal epithelium (Ruthmann et al., 1986; Smith and Reese, 2016). Tight junctions are absent and the adherens junctions provide only a leaky barrier to diffusion (Smith and Reese, 2016). Although cells in epithelia are commonly electrically coupled, no gap junctions are found in *Trichoplax* and its genome lacks genes for connexin and pannexin/innexin-like proteins. Sandwiched between the dorsal and ventral epithelia is a layer of fiber cells with multiple branching processes (Grell and Benwitz, 1971; Buchholz and Ruthmann, 1995; Smith et al., 2014). Fiber cell processes intertwine with epithelial as well as other cell types, but do not form chemical synapses. Fiber cells have been proposed to be contractile, possibly responsible for the amoeboid-like changes in shape of the animal (Grell and Ruthmann, 1991).

Phylogenetic trees inferred from the comparative anatomy of metazoans place Porifera and Placozoa near its base (Harrison and Westfall, 1991; Zrzavý et al., 2005; Schierwater et al., 2009; Nielsen, 2008, 2012). Ctenophora, which have internal digestive tracts, muscles, and complex nervous systems with neurons and synapses, have been thought to be more closely related to Cnidaria and Bilateria. However, several revised phylogenetic trees based on genomic comparisons support the alternative idea that Ctenophora diverged first (Dunn et al., 2008; Hejnol et al., 2009; Ryan et al., 2013; Moroz et al., 2014). The implication of the ‘ctenophore-first’ scenario with regard to the evolution of neurons has been the focus of much discussion. One possibility is that the common ancestor of extant metazoans had neurons that were subsequently lost in Porifera and Placozoa (Ryan and Chiodin, 2015). Another hypothesis is that neurons evolved independently in Ctenophora and Cnidaria/Bilateria (Moroz, 2014; Moroz and Kohn, 2016). Regardless of whether nervous systems evolved once or several times, their precursor was likely to have been a system of coordinated sensory secretory cells (Horridge, 1968; Mackie, 1990; Hartenstein, 2006; Leys and Meech, 2006; Nickel, 2010; Shaham, 2010; Jékely, 2011; Ludeman et al., 2014; Arendt et al., 2015; Leys, 2015; Kristan, 2016).

Many sensory neurosecretory cells in cnidarians and bilaterians are peptidergic (Spencer, 1989; Scharrer, 1990; Cazzamali and Grimmelikhuijzen, 2002; Grimmelikhuijzen et al., 2002; Plickert and Schneider, 2004; Kass-Simon and Pierobon, 2007; Watanabe et al., 2009; Conzelmann et al., 2011; Grimmelikhuijzen and Hauser, 2012; Jékely, 2013; Krishnan and Schiöth, 2015; Caers et al., 2012; Marlow et al., 2014; Arendt et al., 2015; Takahashi and Takeda, 2015). Secretory peptides are produced by cleavage of larger propeptides that include signal peptides for insertion into the secretory pathway through the endoplasmic reticulum and Golgi apparatus, where the propeptides are cleaved and often further processed (Fricker, 2005). Often, secretory peptides have an amidated C-terminus, a modification that may increase their stability and be essential for their activity. Fittingly, the *Trichoplax* genome contains sequences that bear the hallmarks of secretory peptide precursors as well as sequences for the enzymes responsible for propeptide cleavage and amidation (Jékely, 2013; Jekely et al., 2015; Nikitin, 2015). However, *Trichoplax* has never been shown to produce a propeptide for an RFamide-like neuropeptide.

Here, we show that secretory cells in the ventral epithelium of *Trichoplax* package a secretory peptide that arrests ciliary beating during feeding, and that the localized secretion of this peptide is extended to affect ciliated cells across the entire animal, even though the secretory cells are sparse and lack the essential hallmark of a nervous system; namely, long processes that synapse with effector cells.

## MATERIALS AND METHODS

### Materials

#### Animals

*Trichoplax adhaerens* of the Grell (1971) strain, gift of Leo Buss (Yale University), were maintained as described previously (Jackson and Buss, 2009) in artificial seawater (ASW; Instant Ocean, Blacksburg, VA, USA) filtered through a 0.22 µm vacuum filter (no. 431097; Corning, Corning, NY, USA), with *Rhodamonas salina* algae (Bigelow National Center for Culture of Marine Algae and Microbiota, East Boothbay, ME, USA) as a food source. Animals used for experiments were 0.3–1.5 mm in diameter.

#### Peptides

Peptides endomorphin-2 (044-11) and FMRFamide (047-29) were from Phoenix Pharmaceuticals, Inc. (Burlingame, CA, USA). Endomorphin-1 (C-1055) was from Tocris Bioscience (Bristol, UK). Custom-made amidated peptides predicted from *Trichoplax* propeptides (Jékely, 2013; Nikitin, 2015) were RQDYPFF, QDYPFF, DQFFNP, FFNP, QGIPSITF, SITF, QANLKSIFG, SIFG and DQPPRW (Phoenix Pharmaceuticals; and New England Peptide, Inc., Gardner, MA, USA). The non-amidated peptide QDYPFFNG was from NEP (Gardner, MA, USA). Stock solutions (5 mmol l^−1^) in sterile water were stored at −20°C and diluted in ASW before use.

#### Antibodies

Primary antibodies were rabbit anti-endomorphin-2 (H-044-11) and purified anti-endomorphin-2 IgG (G-044-11; Phoenix Pharmaceuticals); custom rabbit antibody against ERNDQRKGYIYWETKC, a sequence from *Trichoplax* endomorphin-2-like propeptide; and custom chicken antibody against CEATAPKKDSSKSNFSSR, a sequence from *Trichoplax* complexin (New England Peptide).

### Methods

#### Identification of the *Trichoplax* endomorphin-like peptide (ELP) transcript

We used a Basic Local Alignment Search Tool (tBLASTn) to search our *Trichoplax* whole-animal mRNA transcriptome for genes bearing endomorphin-like motifs of YPFFG, identifying a single transcript bearing three putative neuropeptide repeats (Fig. S3). To confirm the transcript sequence, we used two separate sets of PCR primers to amplify the full pre-propeptide coding sequence from isolated total RNA via RT-PCR (set 1: TadELP_F1 CATTACAAAATTGCATCACTAGTAAAACTGG, TadELP_F2 TTACCTCAAAAATTACTATTCATGGACC; set 2: TadELP_R1 AACTTCAAACTTCCACTACAATGTCC, TadELP_R2 TTATCCTTTGTAACGATGCTTGTG). The confirmed sequence was submitted to GenBank with accession number KY675296.

#### Time-lapse microscopy of living animals

Animals were rinsed in ASW and placed in a RC-40LP chamber (Warner Instruments, Hamden, CT, USA) containing 0.5 ml ASW. Responses to bath-applied peptide were monitored with transmitted light optics on a LSM510 laser scanning confocal microscope (Carl Zeiss Microscopy, LLC, Thornwood, NY, USA) with 633 nm illumination and a 5× or 10× objective. Peptides were added and mixed into the bath with a handheld pipette. A RSC-200 rapid solution changer (Bio-Logic USA, LLC, Knoxville, TN, USA) was used to apply a ∼200 µl pulse of 5 µmol l^−1^ peptide from a capillary tube positioned ∼1 mm above the animal. The pulse duration was 30 s. A fluorescent dye (CF633; MW∼820; Biotium, Freemont, CA, USA) was added to the peptide solution in some experiments to calibrate the time course of the pulse. The behavior of the animal was monitored with transmitted light on a Nikon Eclipse microscope with a 10× objective. Control experiments consisted of pulse and bath application of ASW without peptide.

The time required for bath-applied peptides to diffuse into the space under an animal was estimated by monitoring diffusion of a fluorescent dye (CF 633) added to the ambient ASW. Fluorescence and transmitted light images were captured with a 40×1.3 NA objective on a Zeiss LSM510 microscope with 633 nm illumination. Fluorescence intensity was compared in two regions, one underneath the animal ∼200 µm from the edge and the other outside the animal.

Images of cilia were captured at 7.6 ms per frame with an Orca 4.0 CMOS camera (Hamamatsu Photonics, Hamamatsu City, Japan) on an Olympus wide-field microscope (Olympus Corporation, Waltham, MA, USA) with 60× NA 1.45 objective, differential interference contrast (DIC) optics and Metamorph image acquisition software (Molecular Devices, Sunnyvale, CA, USA). Calcium-free ASW was made as described previously (Tamm and Tamm, 1981) but supplemented with 2 mmol l^−1^ EGTA.

Time-lapse recordings of animals feeding on algae or pulverized dry algae, cyanobacteria, peptidoglycan, cellulose and charcoal were made with transmitted light and fluorescence optics on a LSM510 with a 10× objective and 543 nm illumination. For monitoring lipophil secretion, FM11-43 dye was added to the medium and fluorescence images were collected with a 40× objective and 488 nm illumination on a Zeiss LSM510 confocal microscope.

#### Immunofluorescence

Animals on amino-silanized coverslips were freeze substituted in tetrahydrafuran on dry ice and fixed in methanol with 1.6% paraformaldehyde as described previously (Smith et al., 2014). Following rehydration in graded dilutions of ethanol and PBS, the samples were transferred to blocking buffer (BB: 3% normal goat serum, 2% horse serum, 1% BSA in PBS) for 15 min. The samples were incubated overnight in primary antibodies and for 3 h in secondary antibodies. Primary antibodies were diluted as follows: anti-endomorphin-2, 1:800; endomorphin-2-like propeptide, 1:400; complexin, 1:100. Secondary antibodies were Atto 488 anti-rabbit (Sigma-Aldrich; 1:500) and Alexa 647 anti-chicken (Thermo Fisher; 1:500). Nuclei were stained with Hoechst dye.

#### Data analysis

Kymographs were constructed from time-lapse image sequences with the montage tool in Metamorph image analysis software. The frequency and duration of periods of gliding and pausing were measured from the kymographs. The animal was considered to be pausing if its edges remained stationary or gradually spread, and dark inclusions in the interior were mostly stationary. These measurements were used to construct bar plots depicting periods of gliding and pausing. Data are expressed as means±s.d. Subtracted images were created with Metamorph software.

#### Imaging immunofluorescence

Fluorescence images were collected on a LSM 880 confocal microscope (Carl Zeiss Microscopy, LLC) with a 40×1.3 NA or 63×1.4 NA plan apo objective with 405, 488 and 633 nm illumination. Overview image stacks were captured with a Quasar spectral detector with emission windows at 415–480 nm (blue) and 490–588 nm (green). Enhanced resolution image stacks were collected with an AiryScan detector and 420–480 nm, 495–550 nm and LP 645 nm filters. Image stacks were displayed as maximum intensity projections.

#### Transmission electron microscopy

Thin-section electron microscopy on freeze-substituted individual *Trichoplax* was performed as previously reported (Smith et al., 2014).

## RESULTS

### A neuropeptide elicits pausing and ciliary arrest

We searched for peptides that cause *Trichoplax* to pause while feeding by obtaining a variety of custom-synthesized peptides encoded by predicted *Trichoplax* propeptides, as well as commercially available peptides resembling predicted *Trichoplax* peptides (Table 1), and adding them to the ASW while monitoring the behavior of the animal by time-lapse bright-field microscopy. Endomorphin-1 (YPWFamide) and endomorphin-2 (YPFFamide) peptides, originally identified in a search for ligands of mu-type opioid receptors in mammalian brain (Zadina et al., 1997), elicited a temporary pause in gliding, and did so even at a concentration as low as 100 nmol l^−1^ (YPFFamide in 3 of 6 animals and YPWFamide in 3 of 3 animals). At concentrations >200 nmol l^−1^, these peptides elicited pausing in 100% of animals (Table 1). In contrast, FMRFamide elicited pausing in 40% of the animals tested, but only at much higher concentrations (>10 µmol l^−1^). One of the tested peptides, SIFGamide, elicited the formation of shallow furrows in the ventral surface at 500 nmol l^−1^ and, at higher concentrations, partial detachment of the animal from the substrate accompanied by folding and writhing.

**Table 1.**
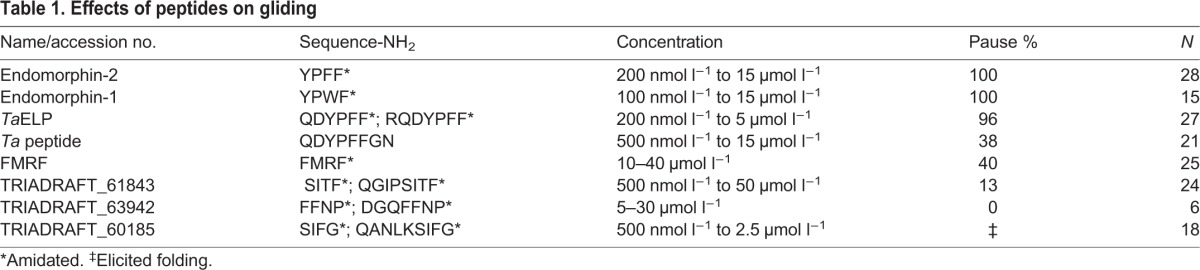
**Effects of peptides on gliding**

None of the propeptide sequences previously identified in *Trichoplax* contained the sequence YPFF, YPWF or FMRF (Jékely, 2013; Nikitin, 2015). However, our ongoing sequencing of the *Trichoplax* whole-animal mRNA transcriptome yielded an assembled transcript encoding a pre-propeptide with three repeats of the sequence Q/EDYPFFGN/S (GenBank accession number KY675296; Fig. S3) each flanked by basic amino acids, the predicted internal convertase cleavage sites required for maturation of neuropeptides from propeptide precursors (Southey et al., 2006). The pre-propeptide sequence bears a predicted N-terminal signal peptide and cleavage site (Petersen et al., 2011) required for targeting of nascent proteins to the secretory pathway. Expression of the KY675296 transcript at the mRNA level was confirmed via RT-PCR amplification from total RNA and Sanger sequencing, as well as by expression level analysis of the transcriptome data with the program RSEM (Li and Dewey, 2011). RSEM alignment of four independently sequenced Illumina RNA-Seq datasets, each representing the *Trichoplax* whole-animal mRNA transcriptome, to their corresponding assembled transcripts produced a transcripts per million expression value of 83±21 for the KY675296 transcript (mean±s.d.; *n*=4), compared with transcripts for reference genes succinate dehydrogenase A (133±8, *n*=4; GenBank accession no. XM_002118121.1) and actin-1 (38±9, *n*=4; GenBank accession no. XM_002107792.1).

**Fig. 1.**
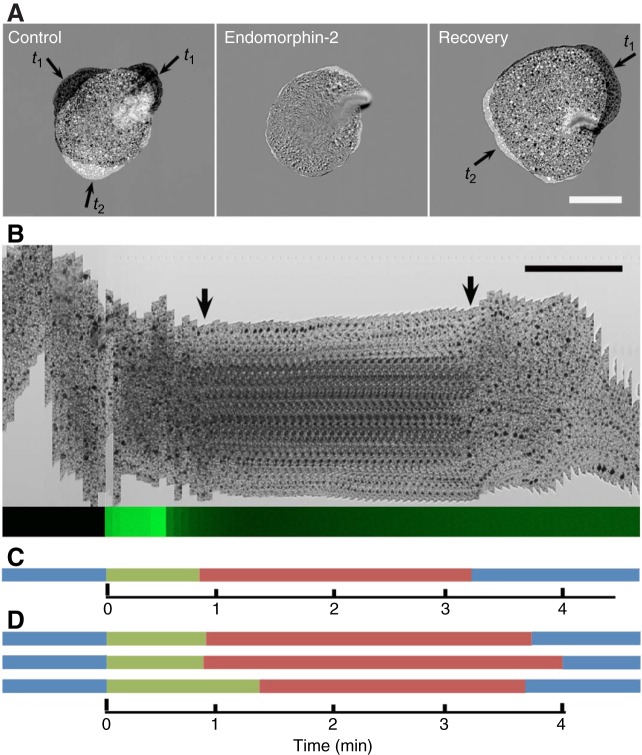
**Endormorphin-2 and** ***Trichoplax* endomorphin-like peptides (*Ta*ELPs) elicit pauses in gliding.** (A) Extent of movement of an animal before (Control), ∼1.5 min after (Endomorphin-2) and 4 min after (Recovery) application of a 30 s pulse of 5 μmol l^−1^ endomorphin-2 from a pipette positioned ∼1 mm above the animal. Subtracted pairs of transmitted light images captured with a confocal microscope are shown. Each image illustrates the silhouettes of the animal at two time points (*t*_1_ and *t*_2_), 19 s apart. Areas around the edge that moved during the 19 s interval appear light or dark, while areas that were stationary match the background gray level. The animal was almost completely stationary 1.5 min after the pulse but had resumed gliding by 4 min. (B) The time courses of pausing and recovery and of the pulse are illustrated by kymographs from transmitted light (top) and fluorescence (bottom) images. The transmitted light kymograph shows sequential images of a narrow (16 µm) region along the vertical axis of the animal captured at 4 s intervals. At the beginning of the sequence, the animal was moving, as evident from the vertical displacement of its edges and dark intracellular inclusions in its interior. Approximately 1 min after the start of the pulse, the animal ceased moving, both its edges and intracellular inclusions becoming mostly stationary (arrows indicate the beginning and end of the pause). The time course of release of peptide was calibrated by monitoring release of a fluorescent dye from the pipette in a separate experiment, depicted by the narrow green kymograph at the bottom. The fluorescence intensity remained high for the 30 s duration of the pulse and dropped rapidly after termination of the pulse. Scale bars in A and B, 200 µm. (C) Gliding (blue) and pausing (red) are plotted in a corresponding bar plot. The green bar indicates the delay between the onset of application of the peptide and the subsequent pause. (D) Three similar bar plots showing effects of release onto three different animals of 5 μmol l^−1^ *Ta*ELP.

**Fig. 2.**
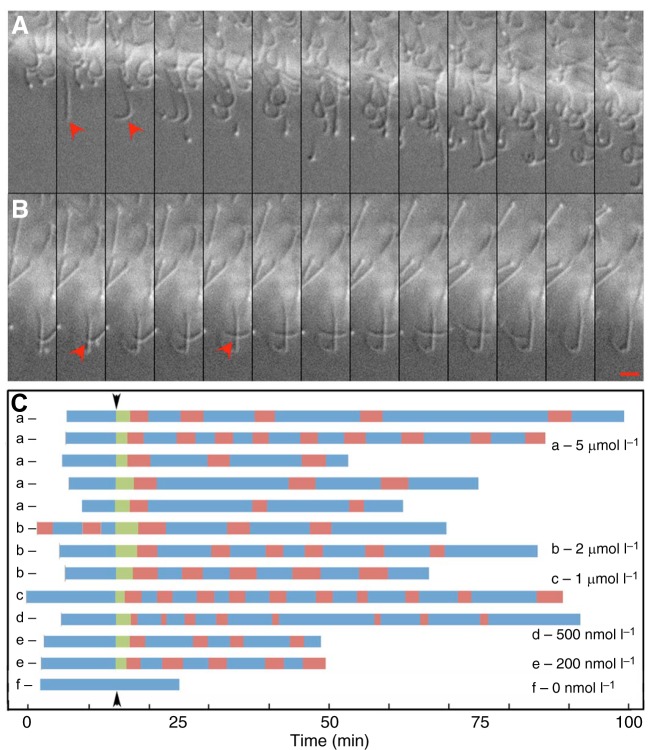
**Bath application of ELPs elicits ciliary** **arrest and repetitive pausing.** (A) Asynchronously beating cilia (red arrowheads) on a gliding animal in differential interference contrast (DIC) images captured at 50 ms intervals. (B) Parallel sequence taken during a pause induced by 1 µmol l^−1^ *Ta*ELP. Most cilia are stationary (red arrowheads). Scale bar, 5 µm. (C) Bar plots showing the effects of bath application of different concentrations of *Ta*ELPs (a–f). Episodes of gliding are depicted in blue and pausing in red. Green bars indicate the delay between the addition of peptide to the bath (starting at the black arrowhead) and the initiation of a pause. The frequency and duration of pauses vary from animal to animal but appear to be independent of concentrations of peptide between 200 nmol l^−1^ and 5 µmol l^−1^. Bath application of artificial seawater (ASW, black arrowhead) without peptide elicited no response.

Custom-synthesized QDYPFFGN, the predicted peptide product following cleavage by convertase, sometimes elicited pausing at 2–5 µmol l^−1^ (38% paused; Table 1: *Ta* peptide). However, QDYPFFamide and RQDYPFFamide, peptides that could be produced if the amino acid following glycine was removed, consistently elicited pausing behavior at >200 nmol l^−1^ (Figs 1 and 2). We focused on these amidated endormorphin-like peptides because of their greater efficacy and will refer to them as *Trichoplax* endomorphin-like peptides (*Ta*ELPs), and when considering them together with endomorphin-1 and -2, as ELPs*.*

The time course of the response to a 30 s pulse of 5 µmol l^−1^ ELPs applied via an injection pipette positioned near the animal was assessed in kymographs created from time-lapse image sequences (Fig. 1). Prior to the addition of ELP, all 15 of the animals examined displayed characteristic behaviors: they ruffled their edges, rotated in place, changed shape and migrated apparently aimlessly. Soon after the addition of ELP, generally within ∼30 s, their movements slowed, and over the next minute or so they became stationary, with almost no movement around their edges and no further movement of the dark particles harbored inside. Then, following a stationary period that lasted 2–4 min, they resumed gliding, at first slowly, but then with increasing speed. Pulse application of ASW without peptide elicited no response (not illustrated).

Bath application of the same peptides produced slower responses than pulse application, adding a delay of 30–60 s, attributable to the time needed for the peptide to mix into the ASW. Movements of the animals began to slow ∼1.5 min after addition of the peptide (Fig. 2). Most of the animals became almost completely stationary at ∼2 min, although the edges of some animals continued to spread gradually to varying extents (not illustrated). The duration of the pauses, defined as the period during which the edges of the animals and the positions of cellular inclusions in their interiors remained almost stationary, was between 2 and 4 min (2.97±0.96 min, *n*=64). Thereafter, the animals resumed gliding, just like the animals that temporarily paused after pulse application of the peptides.

Cilia on the ventral surface beat asynchronously multiple times per second in continuously gliding animals (Fig. 2A; Movie 1). Cilia were mostly stationary but some occasionally beat in paused animals (Fig. 2B; Movie 1). The prevalence of beating versus stationary cilia increased gradually as the animal began to glide again after the termination of the pause (Movie 1).

Animals typically paused only once after pulse application of ELPs. However, they paused repetitively when placed in the continuous presence of the peptides (Fig. 2C). The interval between pauses varied between animals and did not correlate with the concentration of peptide in the bath from 200 nmol l^−1^ to 5 µmol l^−1^ (Fig. 2C). The period between sequential pauses differed between animals, ranging from 5 to 17 min (9.11±4.22 min, *n*=33), but was more uniform within individual animals. Following removal of the peptide and replacement with fresh ASW, the animals no longer paused but moved continuously.

The delay between application of peptides and the initiation of a pause may in part reflect the time required for the peptide to reach the ciliated cells by diffusing into the narrow space between the ventral surface of the animal and the substrate. The diffusion time was estimated by using 1 μm optical sections to visualize the space under gliding animals during bath application of a fluorescent dye (CF 633). The distance between the glass substrate and the ventral surface of the animals was approximately 6 µm, as measured by optical sections in the *z*-axis. The time course of the increase in fluorescence intensity was compared in a region just outside an animal with that in a region underneath the animal, approximately 200 µm from its edge. Animals were roughly circular in shape with a radius of ∼300 µm, and following addition of the dye, the fluorescence intensity reached half-maximal intensity in the interior region 30–80 s later than in the exterior region (49.4±19 s, *n*=10).

### Pausing requires external calcium ions

Ciliary beating typically is regulated by intracellular calcium; ciliary arrest occurs when internal calcium rises beyond a particular level ([Ca^2+^] >10^−4^ mol l^−1^ for mussel gill cells; Walter and Satir, 1978). To determine whether calcium ions are required for pausing behavior in *Trichoplax*, animals were transferred to ASW lacking calcium and containing a calcium chelator prior to application of peptide. Animals maintained in calcium-free ASW remained intact for several hours and their cilia continued beating. They underwent changes in shape similar to those of animals in ASW, but they neither rotated nor glided across the substrate. They were more easily detached from the substrate by the stream of liquid from a pipette than animals in ASW, suggesting that they adhered less strongly to the substrate. Animals in calcium-free ASW did not pause following application of peptides. However, they immediately paused following exchange of about half of the peptide-containing calcium-free ASW with normal ASW, and after pausing, the animals resumed moving and gliding as normal.

### Coordinated gliding and pausing in groups of *Trichoplax*

While observing *Trichoplax* behavior by time-lapse microscopy, we noticed that animals maintained in dishes without food occasionally paused and that when one animal paused, nearby animals typically paused as well. A more detailed investigation of the behavior of animals in groups was undertaken to determine whether diffusion of peptide from a pausing animal could account for the propagation of pausing behavior among animals in groups. Like solitary animals, animals in groups spent much of their time moving: migrating, rotating and changing shape. However, when two or more animals came into contact, they often stayed together for periods ranging from minutes to hours. At sites of contact, the extreme edges of the animals often lifted up from the substrate, bringing together the densely ciliated edges of the ventral epithelia of the adjacent animals. Animals continued to move and change shape while they were in contact, and sometimes separated and then reunited.

**Fig. 3.**
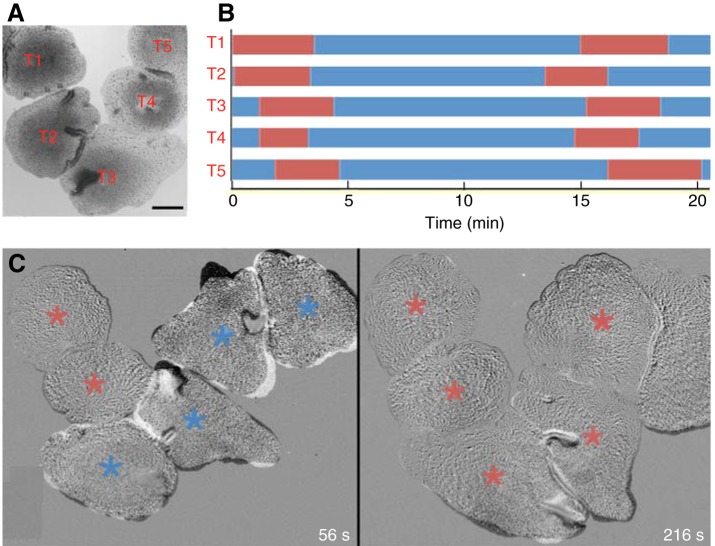
**Pausing behavior propagates from animal to animal.** (A) Transmitted light image documents the positions of five animals (T1–5) tracked in a time-lapse sequence. Scale bar, 200 µm. (B) Bar graphs plot episodes of gliding (blue) and pausing (red). All animals were gliding at the start, but T1 and T2 stopped moving soon after. Animals slowed down and resumed gliding gradually (see Movie 2). (C) Subtracted pairs of images illustrate the extent of movement of the animals during an 8 s interval at two time points after the start of the video. Areas around the edges of animals that moved during the 8 s interval appear light or dark, while areas that were stationary match the background gray level. At 56 s, two animals were stationary (red asterisks) and four were moving (blue asterisks). By 160 s, all six animals were largely stationary although the edges of some spread to varying extents.

Animals in direct contact with other animals manifested pauses in gliding more frequently than solitary animals, which rarely paused in the absence of food (Smith et al., 2015). Pausing behavior spread progressively across members of the group, spanning distances of up to 3 mm, limited only by the field of view (Fig. 3; Movie 2). First, one animal would pause, and then animals in direct contact with the pausing animal paused, followed in turn by animals in contact with those animals. The time at which a member of a group paused was thus directly related to its distance from the first animal that paused (Fig. S1, scatter graph). Even animals separated by a distance of up to ∼200 µm from a group of pausing animals sometimes paused in coordination with the group (Fig. S1). However, animals that were further than 200 µm from a pausing animal did not pause, nor did those that came into the vicinity only shortly before the initiation of the pause. After the animals in the group began moving again, they often separated and changed positions relative to one another. However, they typically reunited and paused again; all 11 groups observed for more than 10 min paused at least twice before dispersing and most groups reunited and paused multiple times. The duration of the pauses was about 4 min (4.13 min, *n*=38 sequential pauses). The average period between the initiation of sequential pauses was about 13 min (12.9±2.0 min, *n*=38). Groups of animals feeding on algae also paused in coordination and the period between sequential pauses was shorter but also more variable (9.09±4.6 min, *n*=38).

### Comparison of pauses elicited by peptide with pauses during feeding

Whether pauses were elicited by peptide (Fig. 1; Fig. S2A) or occurred spontaneously among members of groups (Fig. 2; Fig. S1, Movie 2), they included some, but not all, of the cellular behaviors that accompany feeding on algae (Fig. S2B,C, Movie 3; Smith et al., 2015). Pausing in all circumstances was preceded by a period during which the gliding movements and ruffling around the edges gradually slowed down. Then, within an interval of 10–30 s, the edges of the animal and cellular inclusions in the interior became almost completely stationary and remained so for several minutes. At varying times after the animals ceased moving, their edges gradually spread, and areas in the interior began slow elliptical movements (see Movie 2). Eventually, the animal resumed gliding, sometimes sooner on one side than on the other.

Two components of the behavior during feeding were absent during spontaneous pauses or pauses elicited by application of peptides. During feeding, lipophil cells in the vicinity of algae secreted granules that lysed algae and cells in the vicinity of the lysed algae made undulatory ‘churning’ movements (Smith et al., 2015). These churning movements were evident in kymographs and movies of animals feeding on algae (Fig. S2B, Movie 3). The churning movements were faster and occurred within a smaller area than the subsequent elliptical movements that accompanied resumption of gliding. Churning movements did not occur in animals pausing spontaneously (Movie 2) or after application of peptides, (Fig. 1; Fig. S2A), nor did lipophil cells concomitantly secrete granules, as assessed by direct observation of lipophil granules stained with fluorescent dyes (not illustrated). A role for chemosensing in detecting algae was illustrated by bath application of living red and green microalgae and cyanobacteria, pulverized dry green algae and cyanobacteria, and pulverized peptidoglycan, all of which elicit pausing, churning and lipophil secretion (not illustrated). In contrast, pulverized charcoal or cellulose, particles similar in size to pulverized dry algae, did not elicit pausing.

### Distribution of endomorphin-2 and *Ta*ELP propeptide in secretory cells

**Fig. 4.**
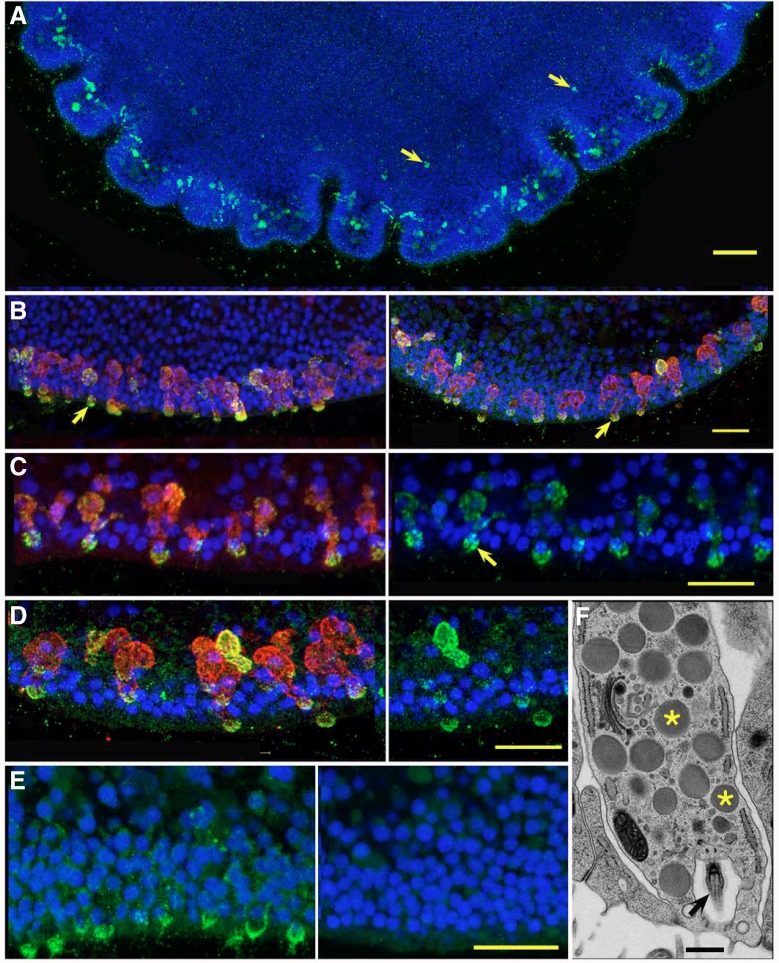
**ELP and *Ta*ELP propeptide are expressed in secretory cells.** Confocal (A–E) and transmission electron microscopy (TEM) (F) images of secretory cells. (A) Enface view of a *Trichoplax* labeled with anti-endomorphin-2. Labeled cells are prevalent around the edge but also present, although dimmer, further toward the center (arrows). (B) Double labeling for endomorphin-2 (left panel; green) or *Ta*ELP propeptide (right panel; green) and complexin (red) in secretory cells near the edge of the animal. Complexin staining extends throughout the cells while endomorphin-2 and *Ta*ELP propeptide staining is concentrated in cell endings at the exterior surface (arrows). (C,D) Enlarged views of cells double-labeled for endomorphin-2 (C, left panel) or *Ta*ELP propeptide (D, left panel) and complexin. Corresponding green channel images (right panels) reveal that label for endomorphin-2 is packaged in larger granules (arrow) than the puncta labeled by the propeptide antibody. (E) Comparison of staining for *Ta*ELP propeptide and matched preimmune control showing lack of *Ta*ELP staining in control. (F) Serial thin section from freeze-substituted animal showing a secretory cell packed with secretory granules (asterisks) and bearing a cilium (arrow) in a deep pocket at the exterior surface. B–D were captured with an AiryScan detector. Scale bars: A, 50 µm; B–E, 10 µm; F, 0.5 µm.

Commercial antiserum against endomorphin-2 (Fig. 4A–C) and purified anti-endomorphin-2 (not illustrated) labeled granules in a population of elongated cells embedded in the columnar epithelium lining the ventral surface of the animal. Corresponding cells in electron micrographs contained many discreet granules 300–500 nm in diameter. These granules were enclosed in membranes, as is typical of peptidergic secretory granules. Most of these cells were arrayed around the circumference near the edge of the animal, spaced ∼5 µm center-to-center (Fig. 4B). Labeled cells were sparse in more central regions of the animal (Fig. 4A). Both the labeled cells around the edge (Fig. 4B,C) and those in the interior (not illustrated) had an extension that reached the surface. Images of labeled granules had dimensions consistent with an actual diameter of ∼400 nm and were distributed throughout the cell body, but concentrated in the endings at the ventral surface. An antibody against complexin, a protein that interacts with secretory SNARE proteins and therefore serves as a marker for neurosecretory cells (Mohrmann et al., 2015; Rizo and Xu, 2015), labeled secretory cells that were stained by the endomorphin-2 antibody as well as some that were not (Fig. 4C), but label for complexin was more uniformly distributed along the long axis of the cell. The close similarity in the appearance and distribution of cells labeled by the endomorphin-2 antibody to that of cells labeled by antibodies against FMRFamide (Smith et al., 2014) or RFamide (Schuchert, 1993) could occur if the antibodies cross-react, recognizing both endomorphin-2 and FMRFamide, as was confirmed in a communication from Phoenix Pharmaceuticals, who provided the anti-endomorphin-2 and FMRFamide antibodies.

We used a custom antibody against an epitope in the putative *Ta*ELP propeptide, distinct from the peptide and secretory signal, to determine whether the cells labeled by the peptide antibodies express the ELP propeptide. Both the antiserum (Fig. 4B,D) and purified antibody (not illustrated) labeled complexin-positive cells arrayed around the edge and sparsely scattered further in the interior. The propeptide label was granular, but the granules appeared smaller than those labeled by the antibodies against endomorphin-2. No cells were labeled by the preimmune serum from the rabbit used for generating the custom antibody against ELP propeptide (Fig. 4E). Examination of serial electron micrographs confirmed that each neurosecretory cell has one cilium of the type with a central pair (Fig. 4F). Cilia originated in pockets on the ventral surface of the neurosecretory cells and lacked the elaborate basal apparatus with an elongated root characteristic of ventral epithelial cells.

## DISCUSSION

*Trichoplax* arrests ciliary beating to cease gliding upon contact with algae, demonstrating that it has a sensory system that detects algae coupled to effectors that exert global control over ciliary beating. We discovered that endomorphin-like peptides (ELPs) consistently elicit cessation of gliding accompanied by ciliary arrest, precisely mimicking pauses that occur during feeding, when applied to the ambient seawater surrounding the animals. Immunolabeling showed that an ELP is packaged in granules in a subset of cells in the ventral epithelium that also express a putative *Trichoplax* ELP (*Ta*ELP) propeptide, as well as complexin, a regulator of SNARE secretory proteins, and voltage-gated calcium channels and SNARE secretory proteins implicated in calcium-regulated secretion (Smith et al., 2014, 2017; Senatore et al., 2016). These secretory cells bear a single cilium distinguished structurally from the motile cilia on epithelial cells, and are therefore likely to be sensory. We propose that these cells are chemosensory neurosecretory cells that upon detection of algae secrete ELP into the ambient seawater, where it directly arrests ciliary beating in the epithelial cells. Their function is analogous to that of sensory neurosecretory cells and neurons that modulate the activity of ciliated cells and other types of effector cells in animals with nervous systems.

Ciliary beating was arrested across the entire ventral epithelium during pauses elicited by contact with algae (Smith et al., 2015), just as it was during pauses elicited by peptide applied to the bath. How could secretion from a relatively small number of ELP-containing secretory cells evoke ciliary arrest throughout the entire animal? The answer we propose comes from the observation that animals in groups paused in coordination. Although animals maintained without food paused infrequently, when one did pause, animals adjacent to it or separated from it by distances up to about 200 µm typically paused soon thereafter. That a signal produced by a pausing animal could evoke pausing in nearby animals is consistent with the idea that the pauses are caused by a chemical signal secreted into the ambient medium, which spreads by diffusion and is attenuated by distance from the source. Moreover, pausing behavior propagated progressively across groups of animals over distances of several millimeters. As the signal produced by an individual pausing animal did not elicit pausing in animals at distances greater than 200 µm, the progressive spread of pausing behavior from animal to animal over millimeters requires that additional diffusible signal must be released in turn by each pausing animal. Because the pausing behavior of these animals was identical to that elicited by application of ELPs, an ELP is likely to be the signal that evokes pausing. A parsimonious explanation of the propagation of pausing behavior from animal to animal is that ELP secreted from each pausing animal evoked ELP secretion in adjacent animals. Propagation of waves of secretion across groups of cells that have receptors for the signal they secrete is well known from work on the social amoeba *Dictyostelium* (Robertson et al., 1972; Devreotes and Steck, 1979; Dormann et al., 2002; Lusche et al., 2005), for instance.

Neurosecretory cells that package ELP are concentrated around the edge of the animal, where the center-to-center spacing is ∼5 µm. Thus, there are ∼1500 ELP-containing secretory cells arrayed around the edge of an animal 500 µm in diameter. Additional secretory cells are scattered in the interior (Smith et al., 2014), but few label for ELP. *Trichoplax* can pause to feed after crawling upon as few as 10 algae. Based on the distribution of cells that immunolabel for ELP, the length of the cilia (∼10 µm) and the size of the algal cells (9×6 µm), we estimate that the number of ELP-containing secretory cells contacting each algal cell is unlikely to exceed five. The granules in thin sections of the neurosecretory cells are 300–500 nm in diameter, twice the volume of the neurosecretory granules in cells of the neural lobe of the pituitary in rats, which contain ∼85,000 molecules of oxytocin (Nordmann and Morris, 1984). If 50 ELP-containing cells that contacted 10 algal cells each secreted one granule containing 170,000 ELP molecules, and the peptide remained in the ∼6 µm space under the animal, the concentration of ELP could reach ∼10 nmol l^−1^. This value is considerably less than the threshold concentration for eliciting a pause by applying ELP in the bath (100 nmol l^−1^), although threshold could be exceeded if each gland cell released 10 granules. Moreover, the concentration of ELP would be further attenuated because the epithelial junctions do not block small molecules from entering the animal from the cleft (Smith and Reese, 2016). Also, the cleft is not occluded at its edge when an animal is gliding, so its contents could diffuse into the ambient seawater.

If ELP secreted by an animal can stimulate secretory cells in adjacent animals to secrete ELP, then it should also stimulate ELP secretion from secretory cells in the same animal. Indeed, the uniform arrest of all cilia throughout the animal during a pause is striking and contrasts with the highly localized control of secretion from lipophils that we interpreted to indicate local signaling pathways (Smith et al., 2015). Ciliary arrest for a pause, after a graded beginning, ultimately affects all cilia. If the ELP secreted by the relatively small number of secretory cells activated by contacting algae triggers the activation of all 1500 secretory cells, then the concentration of ELP in the cleft would reach 360 µmol l^−1^ even if each cell released only one vesicle, a value nearly four times the threshold concentration for eliciting pausing by bath application of peptide. Amplification of the effects of a sensory signal in *Trichoplax* appears to involve a simple positive feedback loop among secretory cells that have autoreceptors for the substance they secrete (Malcangio and Bowery, 1999; Thermos et al., 2006; Taghert and Nitabach, 2012; Bost et al., 2017). An analogous mechanism has been proposed to account for the propagation of waves of contraction across the bodies of cellular sponges (*Demospongiae* and *Calcarea*) when they detect excess particulate matter in their aquiferous canals (Leys and Meech, 2006; Elliott and Leys, 2007). Such positive feedback loops are common in the neuroendocrine systems of bilaterians and help to ensure that once a response is initiated, it proceeds to completion (Brown and Mayeri, 1989; Taghert and Nitabach, 2012).

The *Trichoplax* genome and our *Trichoplax* transcriptome include homologs of eumetazoan peptide-binding G-protein-coupled receptors (GPCRs; Jékely, 2013; Krishnan and Schiöth, 2015; Nikitin, 2015), the most common type of peptide receptor. The time course of minutes, not seconds, for *Trichoplax* to pause and then resume gliding following application of ELP is consistent with ciliary arrest via a GPCR signaling pathway. *Trichoplax* also has homologs of ionotropic RFamide receptors found in *Hydra*. In *Hydra*, these non-selective cationic receptors elicit depolarization and contribute to increased cytosolic calcium levels (Gründer and Assmann, 2015). Conceivably, in *Trichoplax* secretory cells, ELP-sensitive cation receptors could serve to activate voltage-gated calcium channels and ELP exocytosis, and perhaps contribute directly to exocytosis by themselves conducting and increasing cytoplasmic [Ca^2+^]. While we propose that ELP regulates both secretion and ciliary beating, we do not rule out involvement of additional types of transmitters and receptors because *Trichoplax* has sequences for several propeptides in addition to ELP, as well as genes involved in the synthesis and vesicular transport of some classical amino acid neurotransmitters (Srivastava et al., 2008; Alberstein et al., 2015; Krishnan and Schiöth, 2015).

Our observations suggest that when ELP secretory cells detect algae, they secrete ELP, which then elicits secretion of ELP from neighboring cells, leading to a cascade of ELP release. By these means, a small number of secretory cells that detect algal cells can arrest ciliary beating across the entire animal, which can be more than a millimeter in diameter. While a positive feedback cascade extends the distance over which cells can signal by releasing a factor into the ambient medium, the speed of communication over longer distances is limited by the rate of diffusion. Minutes-long periods of pausing and gliding are well suited to a small benthic animal that feeds by external digestion of algae trapped below it. However, to swim and capture faster moving prey, animals ultimately needed a more far-reaching, rapid and specific means of intercellular communication, as achieved by neurons with axons and synapses (Horridge, 1968; Mackie, 1990; Conzelmann et al., 2011; Jékely, 2011).
